# The KAG motif of HLA-DRB1 (β71, β74, β86) predicts seroconversion and development of type 1 diabetes

**DOI:** 10.1016/j.ebiom.2021.103431

**Published:** 2021-06-19

**Authors:** Lue Ping Zhao, George K Papadopoulos, Terry P. Lybrand, Antonis K. Moustakas, George P. Bondinas, Annelie Carlsson, Helena Elding Larsson, Johnny Ludvigsson, Claude Marcus, Martina Persson, Ulf Samuelsson, Ruihan Wang, Chul-Woo Pyo, Wyatt C. Nelson, Daniel E. Geraghty, Stephen S. Rich, Åke Lernmark, the BDD study group

**Affiliations:** a Public Health Sciences Division, Fred Hutchinson Cancer Research Center, 1100 Fairview Ave NE, Seattle, WA 98109, USA; b Laboratory of Biophysics, Biochemistry, Biomaterials and Bioprocessing, Faculty of Agricultural Technology, Technological Educational Institute of Epirus, Arta GR47100, Greece; c Department of Chemistry, Department of Pharmacology and Center for Structural Biology, Vanderbilt University, Nashville, TN, United States; d Department of Food Science and Technology, Faculty of Environmental Sciences, Ionian University, Argostoli GR26100, Cephalonia, Greece; e Department of Clinical Sciences, Lund University, Skåne University Hospital, Lund, Sweden; f Department of Clinical Sciences, Lund University CRC, Skåne University Hospital, Jan Waldenströms gata 35, Skåne University Hospital SUS, Malmö SE-205 02, Sweden; g Crown Princess Victoria Children´s Hospital and Div of Pediatrics, Department of Biomedical and Clinical Sciences, Linköping University, Linköping, Sweden; h Department of Clinical Science and Education Karolinska Institutet and Institution of Medicine, Clinical Epidemiology, Karolinska Institutet, Stockholm, Sweden; i Department of Medicine, Clinical Epidemiological Unit, Karolinska Institutet, Stockholm, Sweden; j Clinical Research Division, Fred Hutchinson Cancer Research Center, Seattle, WA, United States; k Center for Public Health Genomics, University of Virginia, PO Box 800717, MSB Room 3232, 1300 Jefferson Park Ave, Charlottesville, VA 22908, United States

**Keywords:** HLA-DRB1 subtypes, Motif analysis, Seroconversion, Structural analysis, Type 1 diabetes

## Abstract

**Background:**

HLA-DR4, a common antigen of HLA-DRB1, has multiple subtypes that are strongly associated with risk of type 1 diabetes (T1D); however, some are risk neutral or resistant. The pathobiological mechanism of HLA-DR4 subtypes remains to be elucidated.

**Methods:**

We used a population-based case-control study of T1D (962 patients and 636 controls) to decipher genetic associations of HLA-DR4 subtypes and specific residues with susceptibility to T1D. Using a birth cohort of 7865 children with periodically measured islet autoantibodies (GADA, IAA or IA-2A), we proposed to validate discovered genetic associations with a totally different study design and time-to-seroconversions prior to clinical onset of T1D. A novel analytic strategy hierarchically organized the HLA-DRB1 alleles by sequence similarity and identified critical amino acid residues by minimizing local genomic architecture and higher-order interactions.

**Findings:**

Three amino acid residues of HLA-DRB1 (β71, β74, β86) were found to be predictive of T1D risk in the population-based study. The “KAG” motif, corresponding to HLA-DRB1×04:01, was most strongly associated with T1D risk ([O]dds [R]atio=3.64, *p* = 3.19 × 10^−64^). Three less frequent motifs (“EAV”, OR = 2.55, *p* = 0.025; “RAG”, OR = 1.93, *p* = 0.043; and “RAV”, OR = 1.56, *p* = 0.003) were associated with T1D risk, while two motifs (“REG” and “REV”) were equally protective (OR = 0.11, *p* = 4.23 × 10^−4^). In an independent birth cohort of HLA-DR3 and HLA-DR4 subjects, those having the “KAG” motif had increased risk for time-to-seroconversion (Hazard Ratio = 1.74, *p* = 6.51 × 10^−14^) after adjusting potential confounders.

**Interpretations:**

DNA sequence variation in HLA-DRB1 at positions β71, β74, and β86 are non-conservative (β74 A→E, β71 E vs K vs R and β86 G vs V). They result in substantial differences in peptide antigen anchor pocket preferences at p1, p4 and potentially neighboring regions such as pocket p7. Differential peptide antigen binding is likely to be affected. These sequence substitutions may account for most of the HLA-DR4 contribution to T1D risk as illustrated in two HLA-peptide model complexes of the T1D autoantigens preproinsulin and GAD65.

**Funding:**

National Institute of Diabetes and Digestive and Kidney Diseases and the Swedish Child Diabetes Foundation and the Swedish Research Council.

**Research in Context**

Decades of research have shown that type 1 diabetes is an autoimmune disease. The host immune system is wrongfully recognizing specific autoantigens in the pancreatic islet beta cells. The autoantigens are normal constituents and include insulin, GAD65, IA-2 and ZnT8 but when peptides of these autoantigens are presented on beta-cell surface HLA Class I molecules, the otherwise normal beta cells are fatally attacked by self-reactive CD8^+^ cytolytic T cells. These cells are generated by help from CD4^+^ T cells after they recognize autoantigen peptides presented on the surface of Antigen Presenting Cells (APCs). One possible scenario is that beta cells, infected by candidate enterovirus, are dying, engulfed and processed by APC in lymph nodes draining the pancreas. HLA class II heterodimers, encoded by *HLA-DRA-DRB1* or *HLA-DQA1-B1* genes, are known to play essential roles in autoantigen presentation to induce autoreactive CD4^+^ and CD8^+^ T cells as well as B cells producing autoantibodies. The polymorphic HLA class II heterodimers are therefore strongly associated with either risk, neutrality or resistance to the disease. What has been puzzling to all of us is that multiple subtypes of HLA-DRB1*04 molecules play both risk and resistant roles, even though all of them have the same DR4 protein structure. Peeling off complex HLA-DRB1 nomenclature led us to discover responsible amino acids (β71, β74, β86) for this complex “yin-yang” association in a large case-control study. Through investigating the same association in an independent birth cohort, we found that motif “KAG” at these residues was predictive of islet autoimmunity and the first appearing autoantibody. Our finding lays out the foundation for further investigation into the molecular actions between host immune recognition of an environmental factor and the erroneous presentation of autoantigens.

Alt-text: Unlabelled box

## 1 Introduction

*HLA-DRB1* is a class II major histocompatibility (MHC) gene and encodes an antigen-presenting molecule [1]. Together with other MHC II genes (*HLA-DQA1-B1* and *HLA-DPA1-B1*), alleles of the *DRB1* gene are associated with either risk, neutrality or protection of type 1 diabetes (T1D) [2,3]. *HLA* genes are multi-allelic and highly polymorphic, and are associated with many autoimmune diseases, often representing the most significant genetic association with these diseases [4]. There are multiple subtypes also in a single HLA heterodimer such as HLA-DR4. In T1D, the HLA-DR4 subtypes may be associated with risk, neutrality or protection. In Swedish T1D children, we determined by next generation targeted sequencing that the HLA-DR4 had four susceptible subtypes HLA-DRB1*04:01, *04:02, *04:04 and *04:05, two T1D protective subtypes HLA-DRB1*04:03 and *04:07, and one neutral subtype HLA-DRB1*04:08 [5]. Application of the recursive organizer (ROR) identified eleven residues in DRB1, 3, 4 and 5, motifs that capture HLA-DRB1 associations with T1D [6].

The mechanism underlying the associations of HLA-DR4 subtypes with T1D remains largely unknown. The majority of research in T1D cellular autoimmunity has focused on CD4^+^ T cell responses, restricted to HLA-DR4 (particularly HLA-DRB1*04:01) and focused on the four autoantigens (preproinsulin, GAD65, islet antigen (IA)−2, and zinc transporter 8 (ZnT8)) [7]. In the North American and Central-Northern European populations, the *HLA-DR4-DQ8* haplotype is the haplotype most frequently associated with T1D [2,3,8]. There has been one determination of the structure of an HLA-DR4 molecule with a bound T1D-linked autoantigenic peptide, albeit recognized by a regulatory type 1 (Tr1) CD4^+^ T cell [9]. Our systematic analyses have shown that the combination of three residues (β71, β74, β86), all members of anchoring pockets for peptide antigens, are determinants of T1D susceptibility, neutrality or resistance in the various HLA-DR4 molecules detected in the present investigation.

## 2 Methods

### 2.1 Study populations

The current investigation included two studies. The first study is a case-control study which included a total of 962 T1D patients (cases) from the nationwide Swedish Better Diabetes Diagnosis (BDD) study [[10], [11], [12]] and 636 geographically representative controls [13]. These controls are older than patients, and are unlikely to suffer from T1D, since this disease is relatively rare among adults. The second study is The Environmental Determinants of Diabetes in the Young (TEDDY) Study [14,15], designed as a birth cohort. TEDDY included 7865 children with T1D-associated *HLA-DRB1* and *DQA1-B1* haplogenotypes, some (10%) had a first degree relative with T1D. Genetic and follow-up data were obtained from the NIDDK Central Repository (https://repository.niddk.nih.gov/home/), following IRB review of the research protocol. All data elements were described by TEDDY investigators [14,15]. Unlike the BDD study, the TEDDY participants were selected for their HLA increased genetic risks and, therefore, are not representative of any general population.

### 2.2 Ethics

The Karolinska Institute Ethics Board approved the BDD study (2004/1:9). Subjects or their guardian in BDD study provided written consent. De-identified TEDDY data were obtained from NIDDK Central Repository.

### 2.3 Data elements

#### 2.3.1 Phenotype data

**Fig. 1:**
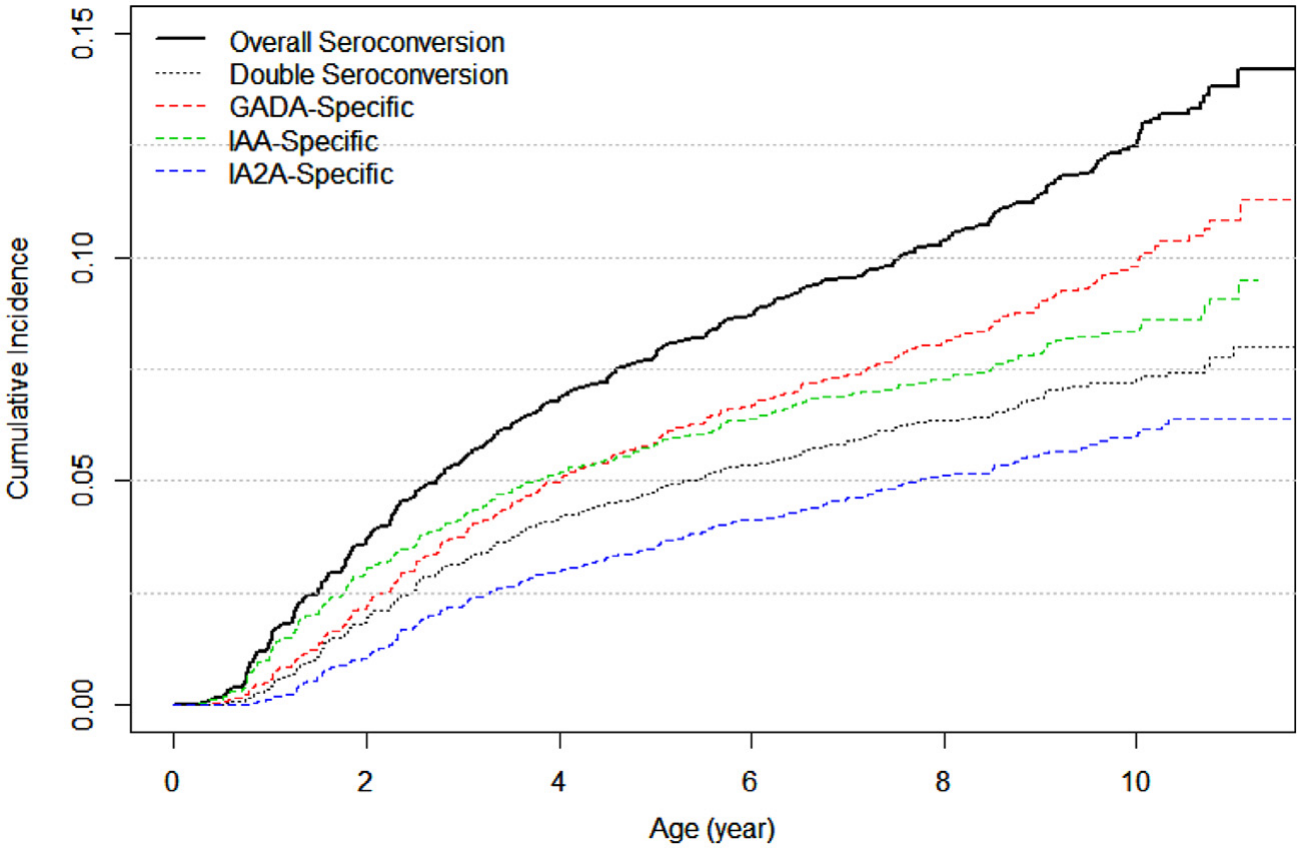
Incidence curves of islet autoantibodies over first decade of boys’ and girls’ lives: overall seroconversion is defined as one or more autoantibodies exceed their respective threshold values (black line), double seroconversion as two or more autoantibodies exceed their respective threshold values (black dotted line), GADA-specific seroconversion as GADA level exceeds its threshold value (red dashed line), IAA-specific seroconversion as IAA level exceeds its threshold value (green dashed line), and IA2A-specific seroconversion as IA2A level exceeds its threshold value (blue dashed line), for the entire cohort.

T1D is a progressively developing disease (**Fig. S1**). The case-control BDD study gathered disease status from patients and selected population-based controls [5]. The TEDDY birth cohort was designed to measure autoantibodies (GADA, IA-2A and IAA) every three months for the first four years following birth and every six months afterwards [16]. These measurements allowed us to construct five different seroconversion events: 1) the first time when any one of three autoantibodies exceeded their threshold values, known as the overall seroconversion, 2) the second time when two or more autoantibodies were elevated, known as the double seroconversion, 3) the first time when GADA exceeded the threshold value, known as GADA-specific seroconversion, 4) IA-2A-specific seroconversion and 5) IAA-specific seroconversion are similarly defined. Their incidence curves were shown in Fig. 1. Erring on the side of caution, our analysis centered on just seroconversion or double seroconversion.

#### 2.3.2 DNA extraction

The plasmid Max isolation kit (Qiagen, Bothell, Washington, USA) was used to isolate DNA according to the manufacturer's instructions from frozen whole blood samples of BDD patients and controls as described [17].

#### 2.3.3 HLA next generation targeted sequencing (NGTS) analysis

The BDD HLA typing was carried using the ScisGo HLA v6 typing kit (Scisco Genetics Inc., Seattle, WA) [17]. Briefly, the method employed an amplicon-based 2-stage PCR, followed by sample pooling and sequencing using a MiSeq v2 PE500 (Illumina, San Diego, CA). The protocol yielded 3-field coverage of all HLA loci including exons 1–4 for DRB1, and genotypes of 3-field were used here. Phase was determined in part by overlapping sequences for HLA class I and database lookup for HLA class II [18]. These types were converted into amino acid sequences, corresponding to those in the beta sheet of codon β1 to β237 as well as those residues in the signal peptide from −29 to −1.

#### 2.3.4 Islet autoantibodies

GADA, IA-2A, IAA, and three variants of ZnT8A (ZnT8-RA, ZnT8-WA or ZnT8-QA) were determined in quantitative radio-binding assays using in-house standards to determine levels as described in details [11].

### 2.4 Statistical analysis methods

#### 2.4.1 Hierarchically organized haplotype (HOH) association analysis

Each DRB1 allele corresponds to a sequence of residues (https://www.ebi.ac.uk/ipd/imgt/hla/), shown for HLA-DRB1*01:01:01 and HLA-DRB1*03:01:01 as well as all subtypes of HLA-DRB1*04 (**Fig. S2**). Appropriately aligned sequences allow us to compute the sequence similarity measurements between all DRB1 alleles, based on which similar allele pairs were clustered closer together, and which were kept apart from those different alleles. All HLA-DRB1 alleles are hierarchically organized and displayed in a tree. Those alleles on the same “tree branch” generally have highly similar residue sequences and, hence, shared the partial HLA nomenclature, *e.g*., HLA-DR4 subtypes fell on the same branch of the hierarchical tree and formed a natural cluster of alleles. Haplotyping individual residues with the cluster membership allowed one to evaluate the residue association within the corresponding cluster.

In the case-control study, association analysis with a binary disease outcome (*y* = 0 for control, and *y* = 1 for patient (or case) used a logistic regression model to assess the outcome association (y) with polymorphic residue (motif, allele/haplotype/genotype) [[19], [20], [21], [22]]. To reduce the challenges of excessive polymorphisms and strong associations with *HLA* loci (e.g. *HLA-DRB1*), we used a “virtual reference” of the null association, and computed odds ratio (OR) as the ratio of a case frequency over a control frequency of the same residue [6]. To evaluate the significance of the estimated odds ratio (OR), we computed haplotype-based score statistic (Z-score) that was used to compute p-values under the assumption of normality. Note that to minimize the distraction with too many statistics, we did not include standard errors or confidence intervals. In case that standard errors are needed for meta-data analysis, one may compute them with estimated OR and Z-score as SE=log(OR)/Z.

In the TEDDY cohort study, association analysis used time-to-seroconversion as an outcome, i.e., a censored outcome. We used the Kaplan-Meier method to estimate the incidence curves [23]. We used a Cox proportional model to assess the association of outcome with genetic polymorphisms as estimated by the hazard ratio (HR) of one polymorphism versus a reference. To eliminate the need of choosing a reference polymorphism, we used a univariate Cox regression analysis of one polymorphism versus all other polymorphisms (combined as a reference). In order to compare risk between polymorphisms, the multivariable Cox regression analysis selected one polymorphism as the reference, while adjusting for potential confounders. From the Cox regression analysis, we computed coefficient, HR (exponentiation of the coefficient), standard error, Z-score, and p-value. Throughout the analysis, we adjusted sex, family history and geographic locations of participants. To ensure the validity of Cox regression result, we tested the proportionality by a Grambsch and Therneau diagnostic test [24], using a R function “cox.zph”.

Presented p-values were unadjusted for multiple comparisons, for the following reasons. Multiple comparisons in the current investigation associated with two study populations, HLA-DR4 subtypes, multiple residues and variable alleles/haplotypes. Corrected p-values by varying numbers of comparisons, by conventional Bonferroni [25] or False Discovery Rate [26], could render inconsistently computed p-values, potentially confusing the interpretation. Further, the current investigation focused on specific residues and motifs of HLA-DR4 subtypes that had well-established disease associations, diminishing the incentive of controlling false positive discoveries.

#### 2.4.2 Molecular simulation of HLA-DR structures not determined by crystallography

Molecular simulation of the structures of HLA-DRB1*04:01, *04:03, *04:04 and *04:05 was carried out as previously described [27], based on the structure of the HLA-DRB1*04:01-collagen complex [28] at ambient pH 5.4. The determination of Insulin A-chain peptides binding to the molecule HLA-DRB1*04:03 has been published previously [29]. The figures drawn for the HLA-DRB1*04:01—Insulin C19-A1 complex were from the respective coordinates of 4y19.pdb, as reported [9]. HLA-DR4 peptide-binding motifs were obtained in [30] and various residue participations in the formation of anchoring pockets are as listed [31]. The properties of well-studied T cell clones restricted to HLA-DR4 molecules and specific for GAD65 555–567 have been previously reported [32,33]. The molecular representations of pMHCII illustrations were drawn with WebLabViewer v. 3.5 (Accelrys, San Diego, CA, USA).

#### 2.4.3 Binding property analysis

Interactive molecular graphics methods were used to assess the possible implications of specific polymorphic substitutions on HLA binding pocket physicochemical properties (e.g., anchor residue binding pocket size, polarity, hydrogen bond donors/acceptors, as well as more general properties such as antigen binding groove electrostatic potential) and the inferred impacts of these physicochemical properties on peptide binding profiles. All analyses were performed using homology-modelled structures noted above.

#### 2.4.4 Statistical functions/package

All data sets were obtained and managed within R studio (https://rstudio.com/) within R system (https://www.r-project.org/). All basic descriptive statistics were generated by R base function. We used the R packages “haplo.stat” (http://cran.r-project.org/web/packages/haplo.stats/index.html) for haplotype analysis and “survival” to compute incidence and log-rank test and to display incidence curves.

### 2.5 Role of the funding sources

Founders and affiliated institutions play no roles in data analysis, result interpretation, and preparation of manuscript.

## 3 Results

### 3.1 Residues β71, β74, and β86 in HLA-DRB1 are responsible for variable associations of HLA-DR4 subtypes with T1D

**Fig. 2:**
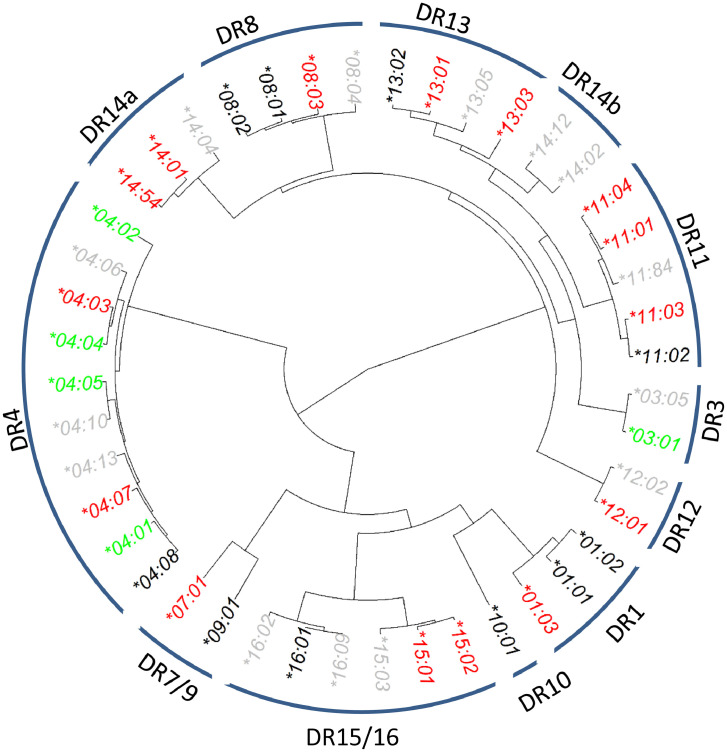
Hierarchically organized DRB1 alleles by similarities of protein sequences observed in 636 controls and 962 patients collected in the BDD case-control study. Alleles are deemed neutral alleles (colored in black font), if their corresponding p-values are greater than 0.05. Otherwise, alleles with estimated odds ratios >1 are considered as risk alleles (alleles colored in green), and those with estimated odds ratios <1 as resistant alleles (colored in red). Alleles with fewer than three copies are deemed to be rare and are colored in gray fonts.

The BDD study included 636 controls and 962 patients and had 44 unique *HLA-DRB1* alleles in this Swedish population. Through hierarchical analysis, multiple clusters of *DRB1* alleles were identified (Fig. 2). We determined the allelic association of T1D with common alleles of *HLA-DRB1* [5]; among the 44 alleles, the *HLA-DR4* cluster included 10 *HLA-DR4* subtypes. Four *HLA-DR4* alleles (HLA-DRB1*04:01, *04:02, *04:04 and *04:05) increased T1D risk (i.e., susceptible) with OR_04:01_ = 3.64 (*p* = 3.19×10^−64^), OR_04:02_ = 2.55 (*p* = 0.025), OR_04:04_ = 1.58 (*p* = 1.91×10^−3^) and OR_04:05_ = 5.95 (*p* = 7.62×10^−4^). Two alleles (HLA-DRB1*04:03 and *04:07) were negatively associated with T1D (i.e., protective) with OR_04:03_ = 0.12 (*p* = 9.18×10^−4^) and OR_04:07_ = 0.11 (*p* = 4.23×10^−4^). One allele (DRB1*04:08) was equal in frequency in T1D cases and controls (*p* = 0.40) (Table 1).

**Table 1:** There are a total of 11 unique DR4 subtypes in the BDD study, and their allelic frequencies (%) among controls and patients are listed. Also listed are their estimated odds ratio, haplotype-score, and p-value. Exluding monomorphic amin acids nets seven amino acids (β37, β57, β67, β70, β71, β74, β86). A p-value is highlighted green, if the odds ratio is significantly greater than *p*=0.05 (without correcting for multiple comparisons). A p-value is highlighted red, if the corresponding odds ratio is significantly less than one. An allele-specific row is colored gray, if it has fewer than three copies. A amino acid colum has a blue fonts if it is invariant among all common alleles.

Upon removing all monomorphic residues within the HLA-DR4 cluster, seven polymorphic residues remained (β37, β57, β67, β70, β71, β74, β86); however, β37 was nearly monomorphic, with a Tyrosine (Y) residue dominating with the exception of a Serine (S) residue on a rare subtype (HLA-DRB1*04:06). Thus, the β37 residue was excluded from additional analyses. Among subjects with HLA-DR4, the association of each residue with T1D was estimated (Table 2). The β57 residue was associated significantly with T1D risk, occurring with aspartic acid (D) or serine (S) (OR_57D_ = 2.56, *p* = 1.43×10^−65^; OR_57S_ = 3.57, *p* = 4.77×10^−3^); however, the OR_57D_ and OR_57S_ were not significantly different (Fisher's exact *p* = 0.66), suggesting that the polymorphic β57 residue is unlikely to explain the T1D association of HLA-DR4 subtypes. Residues β67 and β70 are in complete LD within HLA-DR4 carriers, and the T1D association pattern of β67 and β70 were like that of β57, and unlikely to be critical in the T1D-DR4 subtype effect.

**Table 2:** T1D associations with individual residues, among carriers of DR4: estimated frequencies (%), odds ratio, haplotype-score, p-value and Fisher's eact p-value. A p-value is colored green or red, respectively, if the corresponding odds ratio is significantly greater or less than 1. A p-value corresponding to a residue with two or three amino acid polymorphicsms is highlighted yellow, if T1D association within the residue differs significantly.

Residue β71 in HLA-DR4 was occupied by glutamic acid (E), lysine (K), or arginine (R). Residues E and K were significantly associated with increased T1D risk (OR_71E_ = 2.55, *p* = 0.025; OR_71K_ = 3.61, *p* = 1.22×10^−63^), while residue R had a non-significant (neutral) association with T1D (*p* = 0.642). Residue β86 was occupied by two amino acids glycine (G) and valine (V), both of which were significantly associated with increased T1D risk (OR_86G_ = 3.22, *p* = 3.93×10^−63^; OR_86V_ = 1.41, *p* = 7.68×10^−3^). Residue β74 was occupied by either alanine (A) or glutamic acid (E). The β74A residue was associated with increased T1D risk (OR_74A_ = 2.87, *p* = 1.17×10^−78^) while the β74E residue was associated with decreased T1D risk (OR_74E_ = 0.11, *p* = 5.51×10^−7^). Thus, three amino acid residues (β71, β74, β86) are critical for the association of HLA-DR4 with T1D.

### 3.2 Associated HLA-DR4 motifs with T1D and autoantibodies

The three T1D associated residues (β71, β74, β86) formed seven motifs. Four motifs were associated with increased T1D risk: EAV (OR_EAV_ = 2.55, *p* = 2.47 × 10^−3^), KAG (OR_KAG_ = 3.64, *p* = 3.19 × 10^−4^), RAG (OR_RAG_ = 1.93, *p* = 0.043), and RAV (OR_RAV_ = 1.56, *p* = 2.61 × 10^−3^). Two motifs were associated with decreased T1D risk (REG and REV) with similar effect (OR_REG_ = OR_REV_ = 0.11, *p* = 4.23 × 10^−4^), and one rare motif (KAV) was observed once. The “KAG” motif uniquely corresponded to *HLA-DRB1×04:01*. In addition, a change of the β74 residue from the “RAG” motif to the “REG” motif switched the T1D association from increased risk (OR = 1.93, *p* = 0.043) to very much reduced risk (OR = 0.11, *p* = 4.23 × 10^−4^). Similarly, the paired motifs, “RAV” and “REV”, had similar association patterns.

BDD measured six autoantibodies (GADA, IA-2A, IAA, ZnT8RA, ZnT8WA, ZnT8QA) for all patients at time of the diagnosis. We determined if the four risk motifs (EAV, KAG, RAG and RAV) were associated with autoantibody frequency in patients with HLA-DR4. Autoantibody associations were restricted to GADA and IA-2A (Table 3). The “KAG” motif was significantly associated with IA-2A (OR_KAG_= 2.13, *p* = 1.84 × 10^−17^) but had a reversed association with GADA (OR_KAG_ = 0.83, *p* = 6.32×10^−4^).

**Table 3:** Motif association of (β71, β74, β86) with T1D: estimated frequencies (%) among control and patient, odds ratio, haplotype-score, and p-value. Similarly, motif associations with elevation of GADA and IA2A among patients only: estimated frequencies (%) among those with elevated autoantibody level, odds ratio and p-value. A p-value is highlighted green or red, respectively, if the odds ratio is significantly greater or less than 1.0 .

### 3.3 Variable T1D associations with DRB1 are independent of DQ haplotypes

HLA-DRB1 is in high LD with HLA-DQ haplotypes known to be associated with T1D. We investigated if HLA-DQ haplotypes accounted for the HLA-DR4 subtype-T1D association (Table 4). Haplotypes 3-7 were all HLA-DQA1*03:01-B1*03:02 (DQ8.1) but included five different motifs (EAV, KAG, RAV, REG, REV), each representing HLA-DR4 subtypes with variable T!D associations. Among the four haplotypes in 18–21 (Table 4) sharing the same DQ haplotype, HLA-DQA1*03:03-B1*03:01, the REG motif in HLA-DRB1*04:07 was negatively associated with T1D. On the same DQ haplotype there were two neutral motifs (“KAG” and “RAG”). It is noted that there were 9 DRB1-DQA1-DQB1 haplotypes only present among controls.

**Table 4:** Estimated association result on haplotype association anlysis of HLA-DQA1-B1 with DRB1 motifs (alleles): haplotype count (frequency) among control and patient, estimated odds ratio, Z score and p-value. Blocks of DRB1 motifs are highlighted successively in gray and blue for sharing the same DQ haplotypes.

### 3.4 DRB1 and incidence of seroconversion in the TEDDY cohort

TEDDY screened nearly half million babies to identify ~8000 high-risk babies who had either *HLA-DR3/4, HLA-DR4/4, HLA-DR4/8 or HLA-DR3/3* HLA genotypes (subjects with protective HLA-DRB1×04:03 were excluded, unless they had a positive family history or had other HLA-DR4 alleles in combination with lower risk HLA alleles) [14]. Hence, the TEDDY cohort participants are not representative of the general populations from which they were ascertained (**Table S1** for demographic distribution; **Table S2** for allelic and haplotypic distributions of DRB1, DQA1 and DQB1). Despite the restricted sampling, there were 41 unique HLA-DRB1 alleles. These alleles were hierarchically organized, pointing to a cluster of all HLA-DR4 subtypes associated with T1D risk (**Fig. S3**) with many rare alleles. Three demographic variables (birthplace, family history, sex) associated with seroconversion (**Table S3**) that could confound genetic associations; thus, analyses were adjusted for these three demographic variables. Univariate association of HLA-DR4 subtypes with seroconversion was observed with HLA-DR3 (HLA-DRB1*03:01 and *03:02) and all other alleles combined (Table 5). Univariate Cox analyses assessed the association of one allele compared to all other alleles to show that subjects with *HLA-DRB1×03* had lower risk for seroconversion (HR = 0.85, *p* = 0.014). The strongest association with seroconversion was with HLA-DRB1*04:01 when compared to all non-HLA-DRB1*04:01 alleles (HR = 1.74, *p* = 5.82 × 10^−14^). The HLA-DRB1*04:01 association with seroconversion was consistent for three islet autoantibodies (ZnT8A not available in TEDDY), particularly pronounced for IA-2A-specific seroconversion (HR = 1.90, *p* = 4.63 × 10^−15^).

**Table 5:** Association results from the univariate Cox regression model, assessing DRB1 with seroconversion phenotypes (seroconversion is if two or more autoantibodies exceed the threshold value, overall seroconversion is at least one autoantibody that exceed threshold value, GADA-specific, IAA-specific or IA-2A-specific) among all eligible 7865 subjects from TEDDY cohort: allelic frequency (%), estimated coefficient, hazard ratio, standard error, Z score and p-value, comparing one allele with all other combined as the reference, across all subtypes of DR4 and all other alleles combined. A p-value at 5% without correcting for multiple comparison is used as a threshold. A p-value is highlighted green or red, if it is less than 0.05 and the corresponding HR exceeds one or less than one, respectively. The Cox model adjusts sex, family history and geographic location.

### 3.5 Residues β71, β74, and β86 in HLA-DRB1 are responsible for excessive associations with seroconversion

Monomorphic residues within the HLA-DR4 cluster were excluded, leaving 7 residues, one of which was rare and excluded from further analysis (Table 6). Residues at β67 and β70 were in complete LD; thus, β70 was omitted from analysis. Using the HOH approach, we investigated the association with seroconversion with each residue among HLA-DR4 subjects (Table 7). At β57, aspartic acid (β57D) and serine (β57S) residues had equivalent positive association with seroconversion (HR_57R_ = 1.37, *p* = 3.17 × 10^−5^; HR_57S_ = 1.41, *p* = 0.12). In contrast, at β67, leucine (L) and isoleucine (I) residues differed; β67L was significantly associated with seroconversion while β67I was not (HR_67L_ = 1.43, *p* = 3.33 × 10^−6^; HR_67I_ = 0.97, *p* = 0.89), suggesting that these two residues do not capture the differential effect of HLA-DR4 subtypes. The remaining three residues (β71, β74, β86) were examined for association with seroconversion. Position β71 had three possible amino acids, glutamic acid (E), lysine (K) and arginine (R), with varying direction of effect and significance with seroconversion (HR_71E_ = 0.97, *p* = 0.89; HR_71K_ = 1.74, *p* = 5.93 × 10^−14^; and HR_71R_ = 0.75, *p* = 3.36 × 10^−3^). β74 has either alanine (A) or glutamic acid (E) residues, with opposite association with seroconversion (HR_74A_ = 1.51, *p* = 7.75 × 10^−5^; HR_74E_ = 0.11, *p* = 0.025). Similarly, β86 had two possible residues, glycine (G) and valine (V), with significantly different associations with seroconversion (HR_86G_, *p* = 1.69, *p* = 9.65 × 10^−13^; HR_86V_ = 0.76, *p* = 4.58 × 10^−3^).

**Table 6:** Association analysis from applying the univariate Cox regression model to the TEDDY cohort and assessing association of the time-to-seroconversion with each allele versus all other alleles combined as the reference: estimated frequency (%), coefficient, hazard ratio, standard error, Z-score and p-value, for all alleles in the DR4 cluster. Eliminating all monorphic residues within respective cluster leaves 7 residues. All residues are listed on the right panel. A row is marked in gray, to indicate that the corresponding allelic frequency is less than 10 (rare allele). A colum of residues are marked to have blue font, if it is monomorphic among all common alleles.

**Table 7:** Association result from applying the Cox model to the TEDDY cohort and assessing the association of time-seroconversion with each residue within DR4 cluster: estimated frequency (%) of amino acids, coefficient, hazard ratio, Z-score and p-value, in which one amino acid is compared with all other amino acids in the same cluster. With 5% as the p-value threshold value, a p-value is highlighted red or green, respectively, if the p-value is less than 0.05 and corresponding hazard ratio is less than or greater one, i.e., negatively or positively associated with seroconversion in comparison with all other amino acids. For each residue, the Cox regression model is used to assess differential association of each amino acid with the most common amino acid in their associations with the time to seroconversion, and the association is quantified by the comparison p-values, denoted as Pc. If the Pc is less than 0.05, it is highlighted yellow.

Seven motifs among HLA-DR4 subjects were defined and used in the univariate analysis of association with seroconversion (Table 8). The most significant motif was “KAG”, corresponding to HLA-DRB1*04:01 with a significantly greater association with seroconversion (HR_KAG_ = 1.74, *p* = 5.82 × 10^−14^) than all non-KAG motifs combined. In contrast, the “RAV” motif, corresponding to HLA-DRB1*04:04 and HLA-DRB1*04:10, had a negative association with seroconversion compared with all non-RAV motifs combined (HR_RAV_ = 0.75, *p* = 7.14 × 10^−3^). Two motifs, “KRV” and “KRG”, were grouped (“DR3”), corresponding to two HLA-DR3 alleles, with all other motifs grouped into “OTH”. Univariate analysis (Table 9) demonstrated that the “KAG” motif was significantly associated with seroconversion (HR_KAG_ = 1.74, *p* = 6.51 × 10^−14^). In contrast, subjects with “RAV“, HLA-DR3 or OTH tended to show a negative association with seroconversion. The “KAG” motif, with HLA-DR3 as the reference, had a significant association with seroconversion (HR_KAG_ = 1.68, *p* = 1.96 × 10^−10^). After accounting for all other motifs, subjects with “RAV” and OTH appeared not to differ significantly from HLA-DR3 subjects (RAV-*p* = 0.63; OTH-*p* = 0.14).

**Table 8:** Association results from the univariate analysis of DRB1 motifs of three selected residues (β71, β74, β86) with time-to-seroconversion among DR4 carriers in the TEDDY cohort: estimated frequency (%), coefficient, hazard ratio, standard error, and p-value, when one motif is compared with all other motifs combined. All equivalent HLA-DR4 alleles fulfilling a given motif are listed under “Equivalent”. All amino acids are listed on the far right.

**Table 9:** Association results from the univariate and multivariate association analysis with groups of motifs (DR3, LOW and NEU): Univariate analysis regresses the one motif group versus all others, and multivariate analysis selects one reference motif group (DR3) and estimates their coefficient, hazard ratio, standard error, Z-score, and p-value, in the TEDDY cohort.

**Fig. 3:**
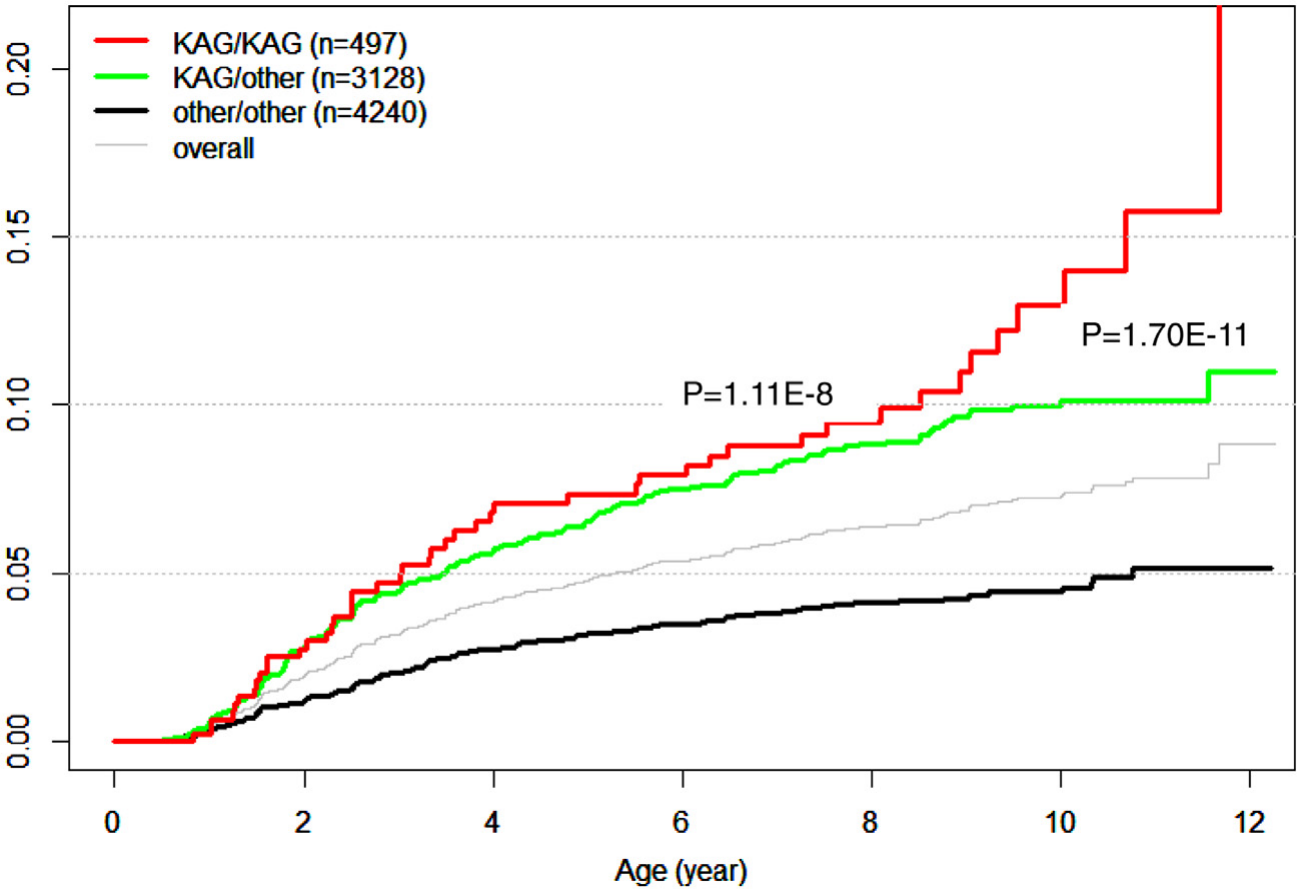
Incidence curves of seroconversion for carriers of homozygous motif “KAG/KAG” (red line), heterozygote “KAG/oth” (green), and all others “oth/oth” (black; motifs combined other than KAG). Both incidence curves among carriers of “KAG” are significantly greater than that of oth/oth (*p* = 1.11×10^−8^ and 1.70×10^−11^, respectively, see Table 10). A thin gray line represents the overall incidence curve.

Grouping the motif “RAV” with HLA-DR3 and OTH, two DRB1 alleles were created: ”KAG” and others. These genotypes were examined for association with seroconversion, including the heterozygote (“KAG/other”) and homozygote (“KAG/KAG”) compared to the homozygote “other/other” as the reference (Table 10). Both the “KAG” heterozygote and homozygote had significantly positive associations with seroconversion (HR_KAG/other_ = 2.07, *p* = 1.70 × 10^−11^; HR_KAG/KAG_ = 2.65, *p* = 1.11 × 10^−8^). Both “KAG” genotypes have significant associations with overall seroconversion, as well as with GADA-specific, IA-2A-specific, and IAA-specific seroconversion. Genotypic associations with IA-2A-specific seroconversion were higher (HR_KAG/other_ = 2.43, *p* = 6.45 × 10^−13^; HR_KAG/KAG_ = 3.07, *p* = 4.04 × 10^−9^), and the KAG homozygote conferred a greater risk than the heterozygote. Cumulative incidence curves for three genotypes (KAG/KAG, KAG/other, and other/other) show that individual homozygous and heterozygous for “KAG” had significantly greater HR for seroconversion than all other genotypes (p_KAG/KAG_ = 1.11 × 10^−8^; p_KAG/other_ = 1.70 × 10^−11^) (Fig. 3). Note that to ensure the robustness of estimating HRs associated with motif genotypes, we tested the assumed proportionality required by the Cox regression model, and found that this assumption was rejected (*p* = 0.408), implying that estimated HRs and related association statistics are appropriate under the proportional hazard model.

**Table 10:** Association results of motif genotypes (KAG/KAG, KAG/other, other/other; other refers to all non-KAG motifs) across five different versions of seroconversions: estimated hazard ratio, Z-score and p-value, treating the homozygous other/other as a reference in the multivariate association analysis of the TEDDY cohort.

## 4 Discussion

The present study represents a novel approach to dissect the contribution of HLA-DR4 in both the etiology and the pathogenesis of T1D. An association between HLA Class I and T1D was reported in The Lancet in 1974 [34] and followed shortly thereafter by the observations that Class II HLA-DR3 and -DR4 were more closely linked to risk of T1D risk than Class I [35]. This was further extended to the linked association with HLA-DQ2 and -DQ8 [36,37], the identification of a single amino acid at HLA-DQ β57 as a strong marker for the risk of T1D [38] and that HLA-DQ6.2 afforded dominant protection [39]. However, further studies are needed to dissect the molecular structures that explain risk and protection from T1D. Our present investigation of HLA-DRB1, centering on HLA-DR4 subtypes, reveals that three residues (β71, β74, β86) are responsible for variable associations of HLA-DR4 subtypes with time-to-seroconversion (reflecting etiology) and T1D (pathogenesis), supported by the two completely independent studies - a birth cohort and a population-based case-control study. The motif “KAG” associates with the time-to-seroconversion (HR = 1.68, *p* = 1.96 × 10^−10^) in the prospectively conducted TEDDY birth cohort. Genotypically, “KAG” had a dose-response relationship with 0, 1 and 2 copies (HR = 1 (reference); HR = 2.07, *p* = 1.70 × 10^−11^; HR = 2.65, *p* = 1.11 × 10^−8^, respectively). In addition, heterozygote and homozygote of “KAG” have stronger association with seroconversion of IA-2A than that of GADA (HR_KAG/other_ = 2.43 vs 1.53 and HR_KAG/KAG_ = 3.07 vs 1.82). It is thus expected that in the BDD case-control study, “KAG” is positively associated with IA-2A (OR_IA2A_ = 2.13) but negatively with GADA OR_GADA_ = 0.83). Additionally, the BDD case-control study indicates that the amino acid β74E corresponds to T1D protection among HLA-DR4 subjects (OR = 0.11, *p* = 5.51 × 10^−7^). Together with residues (β71, β86), a single change from, e.g., “RAV” to “REV”, alters the motifs’ associations from risk to protection (OR_RAV_ = 1.56, *p* = 2.61×10^−3^; OR_REV_ = 0.11, *p* = 4.23 × 10^−4^). As noted, the A → E substitution at β74 alters dramatically the chemical attributes of anchoring pocket p4 and is expected to have a profound impact on antigen peptide binding profiles. While the precise implications for the observed substitutions at β71 (E vs K vs R) and β86 (G vs V) are less obvious, these substitutions also have potentially dramatic impacts on anchor residue preferences via structural modifications they cause in anchor pockets p1, p4 and p7 (and potentially neighboring pocket p6, as seen in the case of HLA-DRB1*04:03^6^). Modeling of specific peptide interactions is needed to address many of these more subtle effects, along with additional experimental investigation as discussed below.

### 4.1 Binding properties of DRB1 residues (β71, β74, β86)

Residue β86 is located near the bottom of the pocket that accommodates peptide anchor residue p1. This residue is either V, or G in the four motifs associated with T1D (EAV, KAG, RAG, and RAV) as well as the two motifs associated with resistance (REG, REV). Either G or V at this position facilitates binding of reasonably large, non-polar p1 anchor residues in this pocket, although G at this position allows for much larger aromatic W, Y, F, p1 anchors. Therefore, G versus V substitution at position β86 may help partially explain variation in antigenic peptide binding preferences that could be correlated with subtle variation in disease progression for different positively associated alleles. More detailed structural studies for specific peptides, will be needed to elucidate the subtle details of G compared to V substitution in the p1 anchor pocket, once the key T1D-associated autoantigenic epitopes, linked to autoimmunity initiation and progression, are identified.

Residue β71 is either glutamic acid (E), lysine (K), or arginine (R) in the positively associated alleles, and arginine (R) in the two resistance alleles. This sequence pattern appears to provide little meaningful information, beyond the observation that this position contributes to a strongly polar profile for the p4 anchor pocket. However, the sequence variation at this position has implication for detailed structure in the p4 anchor pocket. This residue is in contact with neighboring pocket residues, β13H and β28D. The presence of E vs K, or E vs R at this position will alter dramatically the inter-residue interactions, effectively “remodeling” the anchor pocket size and chemical property attributes. The K vs R substitution, sometimes considered as a conservative replacement in protein structures, has dramatic implication for the anchor pocket characteristics. The R residue is much larger than the K residue, but there are important chemical differences as well. The guanidino functional group in the R sidechain can form multiple, highly-directional hydrogen bonds that impose specific structural restraints within this anchor pocket that would not be observed for K at this position. Additionally, local changes in the interactions with β71 and its neighbor residues β13H and β28D may be propagated to additional nearby residues and have impact on anchor pockets p6 and p7. As noted above for position β86, more detailed structural studies with specific peptides will be needed to better understand how E vs K and E vs R at position β71 contribute to differences in antigen binding and disease initiation and progression.

Residue β74 presents the strongest correlation with risk of and resistance to T1D. The substitution of alanine (A) in the disease-associated alleles with glutamic acid (E) in the protective alleles produces a dramatic change in attributes of size, polarity, and hydrogen-bonding of the p4 anchor pocket. The presence of glutamate at this position will preclude binding of many peptides, as the p4 anchor residue cannot be accommodated whether due to size or charge incompatibility. Residue β74 occupancy does not fully explain the T1D risk or protection as positions β71 and β86 also contribute.

### 4.2 Structural considerations from known HLA-DR4 epitopes

**Fig. 4:**
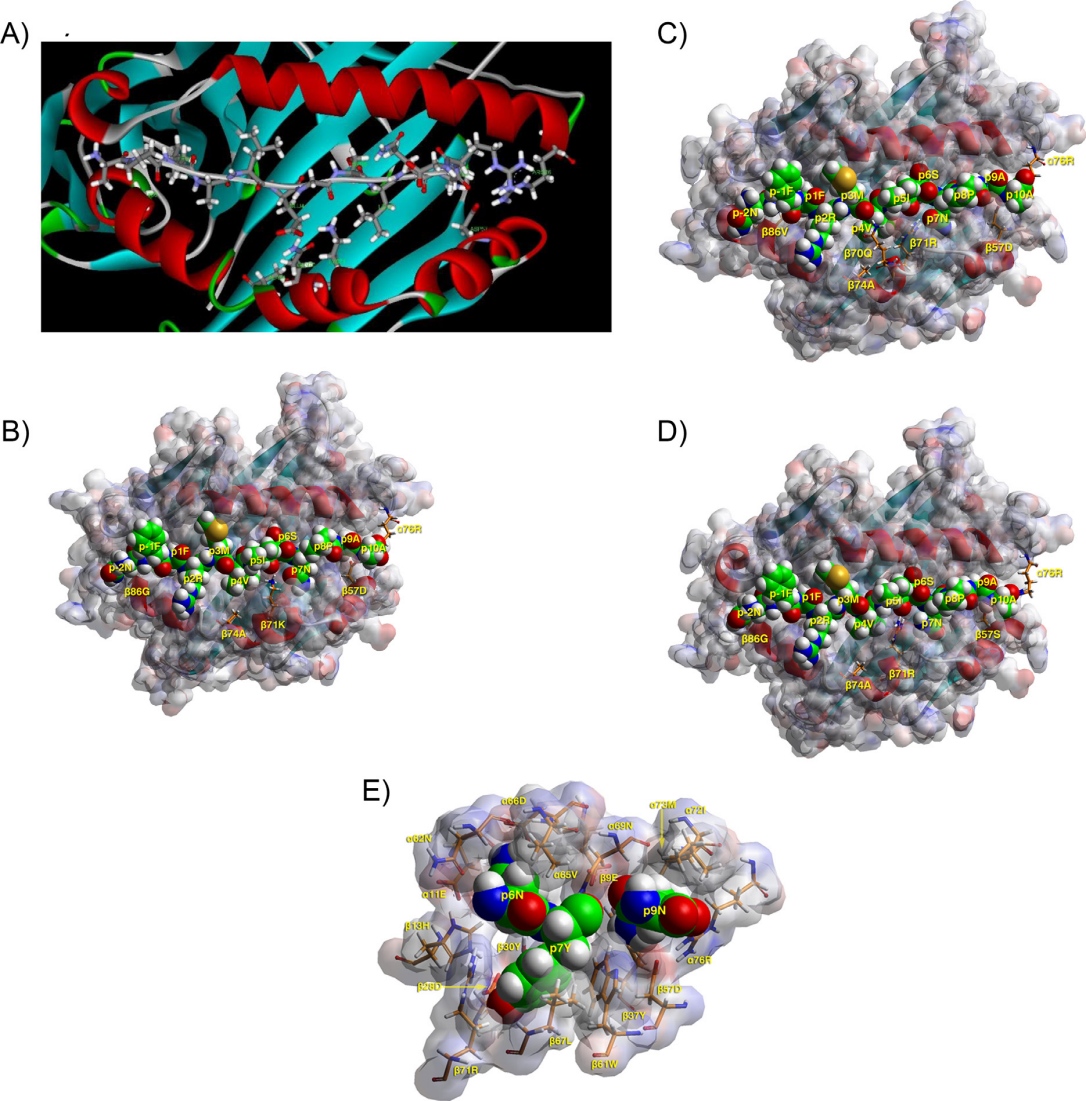
**A)** T Cell Receptor (TCR) view of the DRB1*04:01—InsC19-A1 complex obtained from the deposited coordinates (4y19.pdb) [9]. The α1β1 domain of the MHCII molecule is shown with its secondary structure (α-helix red, β-sheet turquoise, β-turn and random coil in grey), transparent molecular surface colored according to atomic charge (red, negative; blue, positive; gray, neutral, partial charges, in-between colors) and the antigenic peptide in space-filling form (atomic color conventions: carbon, green; oxygen, red; nitrogen, blue; sulfur, yellow; hydrogen, white). Select MHC II residues shown to be important for T1D pathogenesis and seroconversion are shown in stick form (same atom color code, with the exception of carbon that is orange). The structure was obtained in complex with the cognate T cell receptor, hence the turning back of peptide residues p10R and p11G. Anchor p9Lys is perpendicular to the plane of the paper/screen and thus barely seen; the invariant salt bridge between the guanidine group of α76Arg and the carboxylate of β57Asp can be seen in this orientation. **B)** T Cell Receptor (TCR) view of the DRB1*04:01—GAD65 555–566 complex obtained by molecular simulation [31], [32]. **C**) T Cell Receptor (TCR) view of the DRB1*04:04—GAD65 555–566 complex obtained by molecular simulation [33]. **D**) T Cell Receptor (TCR) view of the DRB1*04:05—GAD65 555–566 complex obtained by molecular simulation [33]. Because of the presence of β57S, α76Arg can more easily form a salt bridge with the terminal carboxylate of the antigenic peptide, where in all β57Asp^+^ MHCII alleles α76Arg form a salt bridge with β57Asp. This weaker affinity is indeed seen in the higher IC_50_ value of the same peptide for the DRB1*04:05 molecule [33]. **E)** T Cell Receptor (TCR) view of pockets 6, 7 and 9 of the DRB1*04:03—Ins A11–21 complex obtained by molecular simulation [[6], [28]].

DR4-restricted epitopes to preproinsulin, GAD65, IA-2, and ZnT8 (**Tables S3-S6**) are available as well as reliable autoantibody tests to these autoantigens [7]. Most of the HLA-class II epitopes discovered are probably recognized by TCR on CD4^+^ *T* helper cells, while one such epitope is recognized by a Tr1 regulatory cell (**Table S3**) [7,9]. Structural analysis is focused on those epitopes for which there is extensive characterization of the respective CD4^+^ T cells recognizing the peptide-DR4 complex [7]. The human Ins C19-A1 epitope (LQP**L**AL**E**G**SL**Q**K**RG; anchors in bold) in complex with the HLA-DRB1*04:01 heterodimer fulfills the binding motif for p1, p4 and p6, but less so for p7 and p9 [9,32]. The availability of a crystal structure affords the opportunity of close inspection of the interactions between the peptide and the DRB1×04:01heterodimer [9]. Briefly, p1 has some freedom of movement because of β86 G, and p4 fits snugly interacting electrostatically with β71 K that is positioned between p4E and β28D (Fig. 4A). Likewise, β13H remains positively charged interacting with α11E and β28D. Most often, β71 participates both in pockets 4 and 7, forming the “border” between them [9,30]. Anchor p9K is less favored because of the electrostatic repulsion by α76R in the process of binding; however, once this barrier is overcome, there is substantial attraction from β9E, as well as β35E with interposed water molecules between charged residues that reduce the electrostatic forces [9]. The modelled structure of the DRB1*04:01heterodimer —GAD65 555–567 complex (**Table S3**, Fig. 4B) shows that this epitope (NF**F**RM**V**I**SN**P**A**AT) fulfills the motif of the DRB1*04:01 heterodimer well at all anchor positions [31,32]. This epitope fits well in the same register in the antigen-binding groove of the DRB1*04:04 and DRB1*04:05 heterodimers, despite key differences among DRB1*04:01 and the last two alleles (Fig. 4C and Fig. 4D; Table 1) [31,33]. It also makes it possible to study some of the subtle variations that determine slight differences in IC_50_ peptide binding values, and more so in T cell responses [31,33]. DRB1*04:01 and DRB1*04:05 heterodimers bind the affinity-determining anchor of p1F because of β86 G, while p9A fits best in DRB1*04:01 and DRB1*04:04 because of β57D, instead of β57S in DRB1*04:05. As DRB1*04:04 and DRB1*04:05 heterodimers differ only in β57D/S and β86 V/G, it is not surprising that there are CD4^+^ T cell clones that recognize the GAD65 555–567 peptide in the context of both heterodimers [33]. One such clone extensively tested proliferates better to the native antigen and to various antagonistic altered peptide ligands (APLs), and also secretes more IFN-γ (a Th1 cytokine) and IL-5/IL-13 (Th2 cytokines) in the DRB1*04:04 compared to the DRB1*04:05 heterodimer. The comparison also exhibited differences between the two complexes in their electrostatic surface potentials, something that would indeed play a role in the strength of the associated TCR recognition and subsequent proliferation and cytokine response [33].

The T1D protective DRB1*04:03 and DRB1*04:07 heterodimers, differing from each other only at β86 V/G, respectively, have glutamine (Q) at β70, arginine (R) at β71 and glutamic acid (E) at β74. As shown previously, this generally is expected to forbid acidic or basic residues at p4. In addition, the extensive set of charged interactions β74E^−^••• β71R^+^•••β28D^−^•••β13*H*^+^•••α11E^−^ allow for a polar residue at pockets 6 and 9; the importance and uniqueness of β71R in such interactions has already been stressed [31]. The extensive charge interaction is strengthened by the drawing nearer to pocket 6 of α66D^−^ (Fig. 4E) [6]. As there have not been any CD4^+^ T cell clones or T cell responses restricted to DRB1*04:03 or DRB1*04:07 heterodimers and specific for any T1D autoantigen, we cannot elaborate further on the factors that contribute to T1D resistance by these two β71R/β74E heterodimers.

The analysis of the structural features of the various HLA-DR4 heterodimers and their potential impact on risk of T1D remains incomplete, as we lack the crucial pieces of information regarding the primacy of certain CD4^+^ T cell epitopes in the possible etiological establishment and strengthening of the emerging autoimmune reactions (firstly manifested as seroconversion), and then in the variable length prodrome of pathogenesis finally leading to clinical T1D. Monitoring of antigen-specific epitope spreading in the pre-T1D phase is expensive and time consuming, yet it will be the only way possible in order to answer this question [40]. Consequently, the structural analysis of the various alleles and their role in T1D development is tentative because the detailed characterization of both the etiological and pathogenic roles of most T1D epitopes is missing. For example, the presence of IAA first or GADA first in response to putative environmental triggers may or may not be reflected at the time of clinical onset. The first appearing autoantibody may have been lost during the pathogenic process and second and third appearing autoantibodies taken its place. Other factors of importance may be that HLA-DR4 heterodimers may co-express HLA-DRB4 heterodimers as well. In the case of the Swedish population, we have determined that the vast majority of HLA-DR4^+^ individuals also co-express the DRB4*01:03 heterodimer as well (unpublished). This allele differs from the –DRB4*01:01 allele only in residue β134, whose Gly/Asp-dimorphism has been implicated in T1D pathogenesis (**Fig. S3**) [6]. Thus the antigen-binding motif of –DRB4*01:03 is identical to that of the DRB4*01:01 heterodimer as they have identical antigen-binding α1β1 domains [41]. Future detailed examination of HLA-DR4-restricted epitope spreading should take into account HLA-DRB4-restricted T1D autoantigenic epitopes [41].

Most HLA-DR4 T1D-susceptible heterodimers are also β57D^+^, in contrast to the situation with HLA-DQ molecules [42]. Yet, DRB1*04:05 (β57S) conveys the highest risk for T1D susceptibility. This fact may be related to the presence of β57S instead of the more common D in all other major HLA-DR4 heterodimers. The structural correlates of this substitution have been explained often enough, but bear repeating: lower strength of interaction of β57S with α76R, different preference for p9 anchor residues, different electrostatic surface potential, and thus different rules of selection for cognate TCRs. This might also lead to a lower propensity for the selection of regulatory T cells, an hypothesis that needs to be tested experimentally. The presence of β74E in DRB1*04:03 heterodimers precludes acidic anchoring residues in pocket 4, because of the extensive set of stabilizing charge-charge interactions already outlined (Fig. 4E). Similarly, DRB1*04:04 and DRB1*04:05 heterodimers do not accept basic residues at p4 because of the presence of R, the most basic of residues, at β71 [28,32]. In the only comparative study of the binding epitopes of (prepro)insulin and GAD65 (the two major T1D autoantigens revealed in age-dependent seroconversion in the TEDDY birth cohort [16,43]) to DRB1*04:01, DRB1*04:03 and DRB1*04:05, the latter molecule was shown to bind very few epitopes with IC_50_ value < 10 μM from either antigen (epitope distribution: for preproinsulin, 2/2/0 and for GAD65, 21/23/8, respectively) [29]. This propensity of the DRB1*04:05 heterodimer extended to the H1N1 matrix protein-derived overlapping peptides. Interestingly, it was shown that for 5/6 GAD65 epitopes tested, DRB1*04:03 showed lower rate of dissociation of bound epitope than DRB1*04:01, with comparable IC_50_ values. As HLA-DR4 alleles are in strong LD with HLA-DQ8 (HLA-A1*03:01-B1*03:02), one has to consider the possible effects of epitope stealing between them and the possible effects on T1D etiology rather than pathogenesis [44,45]. This can only be decided by use of specific HLA-DR4-DQ8 antigen-presenting cells and cognate HLA-DR4- or DQ8-restricted T cells that are specific for an overlapping or identical epitope.

It is also unfortunate that there have been to date no DRB1*04:03-restricted CD4^+^ T cell clones (helper or regulatory), specific for any of the major T1D autoantigens. The one available crystal structure of DRB1*04:01—Ins C19-A1 in complex with the F18 TCR of a cognate Tr1 regulatory CD4^+^ T cell does not contain any surprises as far as the pMHC II complex is concerned [9]. As the only example of a human Treg TCR-p-MHC II crystal structure, it shows reverse polarity of TCR binding to pMHC II; this may not be the general mode of TCR binding from Tregs, as two mouse Treg-pMHCII crystal structures were in the conventional orientation [9,46]. The slightly longer proinsulin peptide (C19-A3) that was used in a phase 1b immunotherapy trial of adult newly diagnosed T1D patients seemed to result in higher residual C-peptide compared to those on placebo, no increase in insulin dose, higher FoxP3 expression in CD45RA^−^ Tregs, proinsulin-elicited IL-10 production by CD4^+^ T cells, baseline levels of β-cell specific CD8^+^ T cells, and favorable proinsulin/C-peptide ratio [47]. This underlies the complexity of the disease in that different DR heterodimers may elicit both CD4^+^ T effectors as well as Tregs. It is the overall immune response, together with other T1D susceptibility genes and unknown environmental conditions, that determines β-cell autoimmune response eventually evolving to clinical type 1 diabetes [48,49]. The convenient separation of naïve, effector and regulatory CD4^+^ T cells into various subpopulations by cytometry may demonstrate the relative distribution of epitope-specific T cells into the various subpopulations, with distinct pathogenic properties and roles [50,51].

In the field of autoimmunity, the possibility of certain residues or sequences conferring susceptibility or resistance was put forth nearly 35 years ago [37,52]. Specifically, in the case of rheumatoid arthritis (RA) it was first proposed that susceptibility was linked to a so-called “shared epitope” in relevant HLA-DR4 alleles (mostly), that concerned the β70–74 sequences QKRAA, or QRRAA or RRRAA (i.e. covering residue participating in the formation of pockets 4, 6 and 7) [53]; resistance to the disease was linked to the respective DERAA in resistance-associated HLA-DR alleles [53]. Painstaking research revealed that the RA patients possessing the shared epitope were very likely to have a more severe form of the disease and possess anti-citrullinated protein antibodies (ACPA) targeting citrullinated components of self (e.g. vimentin, aggrecan and others) [53,54]. The mechanism is that peptidyl arginine can be transformed into citrulline (Cit) by the enzyme peptidyl-arginine diimidase (PAD) [55]. Crystallographic and functional studies showed that HLA-DR4 molecules bearing one of the shared-epitope sequences (HLA-DRB1*04:01or *04:04) were unable to bind candidate-epitopes from self-antigens bearing a p4Arg residue [54]. By contrast, these same DR4 molecules could bind the p4Arg→Cit transformed epitope [54]. On the other hand, the protective molecule HLA-DRB1*04:02 heterodimer bearing the DERAA sequence in the β70–74 region, could bind equally well to the relevant citrullinated or native self-epitopes [54]. HLA-DR1*04:01^+^ patients with RA showed increased numbers of aggregan- and viemntin-specific CD4^+^ T cells so restricted, with disease severity correlating to the number of such self-reactive T cells, and a relative lack of Tregs of like specificity and restriction [54]. These findings were verified in the Indigenous North American population where HLA-DRB1*14:02 and HLA–DRB1*04:04 are risk factors, both bearing the shared epitope: the difference is that HLA-DRB1*14:02 having a β13His→Ser-substitution, allows both p4Cit/Arg-anchors in opposite orientations [55]. The ramifications of these findings, in addition to peculiar characteristics of ACPAs and the possibility of putting such knowledge to disease modulation to achieve immunological tolerance was reviewed recently [56,57].

It is concluded from the present study of seroconversion in the TEDDY birth cohort [14,16,43] and of newly diagnosed T1D in the BDD case-control study [6,17] that HLA-DRB1*04 subtypes have a distinct structural motif defined by the three beta-chain amino acid residues HLA-DRB1 (β71, β74, β86). The motif “KAG” was associated with time-to-seroconversion, i.e. most likely reflecting exposures to specific environmental factors that trigger islet autoimmunity. The latter seems to reflect two endotypes, either IAA first or GADA first [16,38]. The association with “KAG” was gene dose dependent as the hazard ratio tended to be higher for two KAG than one. We would speculate that the different motifs observed at β71, β74 and β86 to be associated with an increased, neutral or decreased risk of T1D are reflected in an environmental factor presented differently on the DRB1*04 subtype heterodimer. For example, prolonged enterovirus B infection was associated with an increased risk for IAA as the first detected autoantibody, but not with GADA as the first islet autoantibody, in children younger than three years of age [58]. In older children, other common enterovirus infections were related to GADA as the first detected autoantibody. Although the analysis of autoantigen peptide binding to different HLA-DRB1*04 subtypes are likely crucial to the subsequent pathogenesis, the importance of, e.g. virus antigen peptide binding to the different DRB1*04 subtypes should not be overlooked. It may be that understanding a possible interaction between triggering virus or other antigen and competing autoantigen presentation on susceptible, neutral or protective HLA-DRB1*04 subtype heterodimers may reveal mechanisms of HLA-associated organ-specific autoimmune diseases such as T1D [59].

## Declaration of Competing Interest

Dr. Claude Marcus receives personal fees from Novo Nordisk and there are no other conflicts of interest relevant to this article. Other authors report no conflict of interest.

## Appendix Supplementary materials

Image, application 1

Image, application 2

Image, application 3

Image, application 4

## References

[1] Robinson J., Halliwell J.A., Hayhurst J.D., Flicek P., Parham P., Marsh S.G. (2015). The IPD and IMGT/HLA database: allele variant databases. Nucleic Acids Res.

[2] Noble J.A., Valdes A.M. (2011). Genetics of the HLA region in the prediction of type 1 diabetes. Curr Diab Rep.

[3] Erlich H., Valdes A.M., Noble J. (2008). HLA DR-DQ haplotypes and genotypes and type 1 diabetes risk: analysis of the type 1 diabetes genetics consortium families. Diabetes.

[4] Dendrou C.A., Petersen J., Rossjohn J., Fugger L. (2018). HLA variation and disease. Nat Rev Immunol.

[5] Zhao L.P., Alshiekh S., Zhao M. (2016). Next-generation sequencing reveals that HLA-DRB3, -DRB4, and -DRB5 may be associated with islet autoantibodies and risk for childhood type 1 diabetes. Diabetes.

[6] Zhao L.P., Papadopoulos G.K., Kwok W.W. (2019). Eleven amino acids of HLA-DRB1 and fifteen amino acids of HLA-DRB3, 4, and 5 include potentially causal residues responsible for the risk of childhood type 1 diabetes. Diabetes.

[7] James E.A., Mallone R., Kent S.C., DiLorenzo T.P. (2020). T-cell epitopes and neo-epitopes in type 1 diabetes: a comprehensive update and reappraisal. Diabetes.

[8] Pociot F., Lernmark A. (2016). Genetic risk factors for type 1 diabetes. Lancet.

[9] Beringer D.X., Kleijwegt F.S., Wiede F. (2015). T cell receptor reversed polarity recognition of a self-antigen major histocompatibility complex. Nat Immunol.

[10] Delli A.J., Lindblad B., Carlsson A. (2010). Type 1 diabetes patients born to immigrants to Sweden increase their native diabetes risk and differ from Swedish patients in HLA types and islet autoantibodies. Pediatr Diabetes.

[11] Delli A.J., Vaziri-Sani F., Lindblad B. (2012). Zinc transporter 8 autoantibodies and their association with SLC30A8 and HLA-DQ genes differ between immigrant and Swedish patients with newly diagnosed type 1 diabetes in the Better Diabetes Diagnosis study. Diabetes.

[12] Carlsson A., Kockum I., Lindblad B. (2012). Low risk HLA-DQ and increased body mass index in newly diagnosed type 1 diabetes children in the Better Diabetes Diagnosis study in Sweden. Int J Obes.

[13] Gyllenberg A., Asad S., Piehl F. (2012). Age-dependent variation of genotypes in MHC II transactivator gene (CIITA) in controls and association to type 1 diabetes. Genes Immun..

[14] Hagopian W.A., Erlich H., Lernmark A. (2011). The Environmental Determinants of Diabetes in the Young (TEDDY): genetic criteria and international diabetes risk screening of 421 000 infants. Pediatr Diabetes.

[15] Group T.S. (2008). The environmental Determinants of Diabetes in the Young (TEDDY) study. Ann N Y Acad Sci.

[16] Krischer J.P., Lynch K.F., Schatz D.A. (2015). The 6 year incidence of diabetes-associated autoantibodies in genetically at-risk children: the TEDDY study. Diabetologia.

[17] Zhao L.P., Alshiekh S., Zhao M. (2016). Next-generation sequencing reveals that HLA-DRB3, -DRB4, and -DRB5 may be associated with islet autoantibodies and risk for childhood type 1 diabetes. Diabetes.

[18] Nelson W.C., Pyo C.W., Vogan D. (2015). An integrated genotyping approach for HLA and other complex genetic systems. Hum Immunol.

[19] Lake S.L., Lyon H., Tantisira K. (2003). Estimation and tests of haplotype-environment interaction when linkage phase is ambiguous. Hum Hered..

[20] Schaid D.J., Rowland C.M., Tines D.E., Jacobson R.M., Poland G.A. (2002). Score tests for association between traits and haplotypes when linkage phase is ambiguous. Am J Hum Genet.

[21] Li S.S., Cheng J.J., Zhao L.P. (2007). Empirical vs Bayesian approach for estimating haplotypes from genotypes of unrelated individuals. BMC Genet..

[22] Zhao H., Pfeiffer R., Gail M.H. (2003). Haplotype analysis in population genetics and association studies. Pharmacogenomics.

[23] Kaplan E.L., Meier P. (1958). Nonparametric estimation from incomplete observations. J Am Stat Assoc.

[24] Grambsch P.M., Therneau T.M. (1995). Proportional hazards tests and diagnostics based on weighted residuals (Vol 81, Pg 551, 1994). Biometrika.

[25] Benjamini Y., Hochberg Y. (1995). Controlling the false discovery rate: a practical and power approach to multiple testing. JRSS B.

[26] Storey J.D. (2002). A direct approach to false discovery rates. J R Stat Soc Ser B.

[27] Reichstetter S., Papadopoulos G.K., Moustakas A.K. (2002). Mutational analysis of critical residues determining antigen presentation and activation of HLA-DQ0602 restricted T-cell clones. Hum Immunol.

[28] Dessen A., Lawrence C.M., Cupo S., Zaller D.M., Wiley D.C. (1997). X-ray crystal structure of HLA-DR4 (DRA*0101, DRB1×0401) complexed with a peptide from human collagen II. Immunity.

[29] Ge X., James E.A., Reijonen H., Kwok W.W. (2011). Differences in self-peptide binding between T1D-related susceptible and protective DR4 subtypes. J Autoimmun.

[30] Bondinas G.P., Moustakas A.K., Papadopoulos G.K. (2007). The spectrum of HLA-DQ and HLA-DR alleles, 2006: a listing correlating sequence and structure with function. Immunogenetics.

[31] Reijonen H., Novak E.J., Kochik S. (2002). Detection of GAD65-specific T-cells by major histocompatibility complex class II tetramers in type 1 diabetic patients and at-risk subjects. Diabetes.

[32] Friede T., Gnau V., Jung G., Keilholz W., Stevanovic S., Rammensee H.G. (1996). Natural ligand motifs of closely related HLA-DR4 molecules predict features of rheumatoid arthritis associated peptides. Biochim Biophys Acta.

[33] Masewicz S.A., Papadopoulos G.K., Swanson E., Moriarity L., Moustakas A.K., Nepom G.T. (2002). Modulation of T cell response to hGAD65 peptide epitopes. Tissue Antigens.

[34] Nerup J., Platz P., Andersen O.O. (1974). HL-A antigens and diabetes mellitus. Lancet.

[35] Platz P., Jakobsen B.K., Morling N. (1981). HLA-D and -DR antigens in genetic analysis of insulin dependent diabetes mellitus. Diabetologia.

[36] Owerbach D., Lernmark A., Platz P. (1983). HLA-D region beta-chain DNA endonuclease fragments differ between HLA-DR identical healthy and insulin-dependent diabetic individuals. Nature.

[37] Todd J.A., Bell J.I., McDevitt H.O. (1987). HLA-DQ beta gene contributes to susceptibility and resistance to insulin-dependent diabetes mellitus. Nature.

[38] Morel P.A., Dorman J.S., Todd J.A., McDevitt H.O., Trucco M. (1988). Aspartic acid at position 57 of the HLA-DQ beta chain protects against type I diabetes: a family study. Proc Natl Acad Sci USA.

[39] Baisch J.M., Weeks T., Giles R., Hoover M., Stastny P., Capra J.D. (1990). Analysis of HLA-DQ genotypes and susceptibility in insulin-dependent diabetes mellitus. N Engl J Med.

[40] Vaziri-Sani F., Delli A.J., Elding-Larsson H. (2011). A novel triple mix radiobinding assay for the three ZnT8 (ZnT8-RWQ) autoantibody variants in children with newly diagnosed diabetes. J Immunol Methods.

[41] James E.A., Gillette L., Durinovic-Bello I. (2018). DRB4*01:01 has a distinct motif and presents a proinsulin epitope that is recognized in subjects with type 1 diabetes. J Immunol.

[42] Zhao L.P., Papadopoulos G.K., Kwok W.W. (2020). Motifs of three HLA-DQ amino acid residues (alpha44, beta57, beta135) capture full association with the risk of type 1 diabetes in DQ2 and DQ8 children. Diabetes.

[43] Krischer J.P., Liu X., Vehik K. (2019). Predicting islet cell autoimmunity and type 1 diabetes: an 8-year TEDDY study progress report. Diabetes Care.

[44] Ge X., Piganelli J.D., Tse H.M. (2006). Modulatory role of DR4- to DQ8-restricted CD4 T-cell responses and type 1 diabetes susceptibility. Diabetes.

[45] Vehik K., Bonifacio E., Lernmark A. (2020). Hierarchical order of distinct autoantibody spreading and progression to type 1 diabetes in the TEDDY study. Diabetes Care.

[46] Stadinski BD, Blevins SJ, Spidale NA (2019). A temporal thymic selection switch and ligand binding kinetics constrain neonatal Foxp3^+^ T_reg_ cell development.. Nature Immunology.

[47] Alhadj Ali M., Liu Y.F., Arif S. (2017). Metabolic and immune effects of immunotherapy with proinsulin peptide in human new-onset type 1 diabetes. Sci Transl Med.

[48] Wen X., Yang J., James E., Chow I.T., Reijonen H., Kwok W.W. (2020). Increased islet antigen-specific regulatory and effector CD4(+) T cells in healthy individuals with the type 1 diabetes-protective haplotype. Sci Immunol.

[49] Habib T., Long S.A., Samuels P.L. (2019). Dynamic immune phenotypes of B and T helper cells mark distinct stages of T1D progression. Diabetes.

[50] Miyara M., Yoshioka Y., Kitoh A. (2009). Functional delineation and differentiation dynamics of human CD4+ T cells expressing the FoxP3 transcription factor. Immunity.

[51] Petsiou A., Paschou S.A., Vartholomatos G. (2020). A modified flow cytometry method for objective estimation of human CD4. Cytometry B Clin Cytom.

[52] Gregersen P.K., Silver J., Winchester R.J. (1987). The shared epitope hypothesis. An approach to understanding the molecular genetics of susceptibility to rheumatoid arthritis. Arthritis Rheum.

[53] Kampstra A.S.B., Toes R.E.M. (2017). HLA class II and rheumatoid arthritis: the bumpy road of revelation. Immunogenetics.

[54] Scally S.W., Petersen J., Law S.C. (2013). A molecular basis for the association of the HLA-DRB1 locus, citrullination, and rheumatoid arthritis. J Exp Med.

[55] Scally S.W., Law S.C., Ting Y.T. (2017). Molecular basis for increased susceptibility of Indigenous North Americans to seropositive rheumatoid arthritis. Ann Rheum Dis.

[56] Toes R., Raza K. (2021). Therapeutic tolerance induction 3: the autoimmune response as a potential target for tolerance induction before the development of rheumatoid arthritis. Lancet Rheumatol.

[57] Koga T, Kawakami A, Tsokos GC (2021). Current insights and future prospects for the pathogenesis and treatment for rheumatoid arthritis.. Clin Immunol.

[58] Vehik K., Lynch K.F., Wong M.C. (2019). Prospective virome analyses in young children at increased genetic risk for type 1 diabetes. Nat Med.

[59] Fugger L, Jensen LT (2020). Challenges, Progress, and Prospects of Developing Therapies to Treat Autoimmune Diseases.. Cell.

