# Supplementary material for: The KAG motif of HLA-DRB1 (β71, β74, β86) predicts seroconversion and development of type 1 diabetes
Source: eBioMedicine. 2021 Jun 19;69:103431. doi: 10.1016/j.ebiom.2021.103431 (PMC8220560; doi:10.1016/j.ebiom.2021.103431)
Supplement: Supplementary file 1 [file mmc1.docx]

**Supplementary Figures**


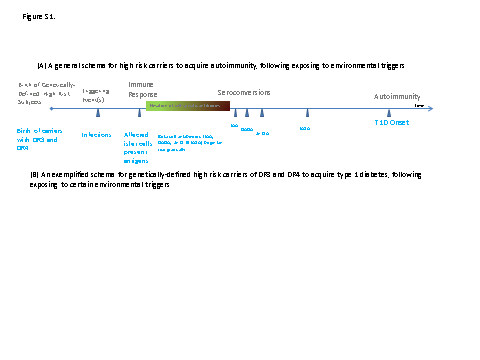


Figure S1. An illustration on how a carrier of high risk genetic factors may expose to environmental triggers, the host immune system responses to the triggers, cell- or tissue-specific antibodies are produced by the immune system, excessive elevations of one or more autoantibodies lead to seroconversion, and ultimately the host immune system starts to destroy the own healthy cells or tissues: (A) represents a general schema and (B) a schema specifically illustrated for type 1 diabetes

**Fig. S2 Select HLA-DR4/-DRB4 amino acid sequences**

**10 20 30 40 50 60 70 80 90**

**DRB1*01:01:01 GDTRPRFLWQLKFECHFFNGTERVRLLERCIYNQEESVRFDSDVGEYRAVTELGRPDAEYWNSQKDLLEQRRAAVDTYCRHNYGVGESFT**

**DRB1*03:01:01 ________EYSTS____________Y_D_YFH____N_________F_______________________K_GR__N________V____**

**DRB1*04:01:01 ________E_V_H____________F_D_YF_H___Y_________________________________K___________________**

**DRB1*04:02 ________E_V_H____________F_D_YF_H___Y_____________________________I__DE______________V____**

DRB1*04:03:01 ________E_V_H____________F_D_YF_H___Y____________________________________E___________V____

**DRB1*04:04 ________E_V_H____________F_D_YF_H___Y________________________________________________V____**

**DRB1*04:05:01 ________E_V_H____________F_D_YF_H___Y___________________S_________________________________**

DRB1*04:06:01 ________E_V_H____________F_D_YF_H________________________________________E___________V____

DRB1*04:07:01 ________E_V_H____________F_D_YF_H___Y____________________________________E________________

DRB1*04:08 ________E_V_H____________F_D_YF_H___Y_____________________________________________________

DRB1*04:10 ________E_V_H____________F_D_YF_H___Y___________________S____________________________V____

DRB1*04:13 *****___E_V_H____________F_D_YF_H___Y_________________________________K______________V____

DRB4*01:01 ___Q____E_A_C____L______WN_I_Y______YA_YN__L___Q_____________________R___E______Y____V____

DRB4*01:03 ___Q____E_A_C____L______WN_I_Y______YA_YN__L___Q_____________________R___E______Y____V____

9 6 4 **|** **~**4 4 6 9 **~** 7 @@@@@@@ 9 7 7 44 4 **~** 4**|~**!! 11 11

**| +** 7 ! **-** ! ! 77 **- |+**

**| |_________________| |__||**

**|_______________________________S-S_____________________________|**

**91 100 110 120 130 140 150 160 170 180**

**DRB1*01:01:01 VQRRVEPKVTVYPSKTQPLQHHNLLVCSVSGFYPGSIEVRWFRNGQEEKAGVVSTGLIQNGDWTFQTLVMLETVPRSGEVYTCQVEHPSV**

**DRB1*03:01:01** _____H___________________________________________T________H_______________________________

**DRB1*04:01:01 _____Y_E_____A_______________N___________________T_______________________________________L**

**DRB1*04:02 _____Y_E_____A_______________N___________________T_______________________________________L**

DRB1*04:03:01 _____Y_E_____A_______________N___________________T_______________________________________L

**DRB1*04:04 _____Y_E_____A_______________N___________________T_______________________________________L**

**DRB1*04:05:01 _____Y_E_____A_______________N___________________T_______________________________________L**

DRB1*04:06:01 _____Y_E_____A_______________N___________________T_______________________________________L

DRB1*04:07:01 _____Y_E_____A_______________N___________________T_______________________________________L

DRB1*04:08 _____Y_E_____A_______________N___________________T_______________________________________L

DRB1*04:10 _____Y_E_____A_______________N___________________T_______________________________________L

DRB1*04:13 ******************************************************************************************

DRB4*01:01 _____Q_______________________N______________S____________________________________________M

DRB4*01:03 _____Q_______________________N___________________________________________________________M

# ## # **|** /////////////// # **|**

**|** ## **|**

**|________________________S-S____________________________|**

**Fig. S2 Select HLA-DR4/-DRB4* amino acid sequences (continued)**

**181 190 200 210 220 230 237**

**DRB1*01:01:01** **TSPLTVEWRARS**ESAQSKMLSGVGGFVLGLLFLGAGLFIYFRNQKGHSGLQPTGFLS

**DRB1*03:01:01** **____________**________________________________________R____

**DRB1*04:01:01** **____________**_____________________________________________

**DRB1*04:02 ____________**_____________________________________________

DRB1*04:03:01 _________________________________________________________

**DRB1*04:04 ____________**_____________________________________________

**DRB1*04:05:01** **____________**_____________________________________________

DRB1*04:06:01 _________________________________________________________

DRB1*04:07:01 _________________________________________________________

DRB1*04:08 _________________________________________________________

DRB1*04:10 _________________________________________________________

DRB1*04:13 *********************************************************

DRB4*01:01 M_____Q_S_________________________T___________________L__

DRB4*01:03 M_____Q_S_________________________T___________________L__

Notes :

1. Alleles with known crystal structure are in bold.

2. Identity in residues is indicated by _, while unknown residues are shown as *.

3. Symbols below the sequence of the last allele:

For antigen binding (highlighted in yellow): residues participating in the formation of a particular pocket indicated by the number of the pocket (1, 4, 6, 7, 9) at the bottom of the column; in case a residue participates in more than one pockets this is indicated by numbers on two lines. For interchain interactions: ! ; residues forming hydrogen bonds with antigenic peptide backbone (highlighted in red); ~: intrachain salt bridges marked with respective charge signs are highlighted in blue. # : residues participating in the formation of the homodimer of heterodimers (highlighted in pink); / : residues involved in the binding of CD4 (highlighted in turquoise). Disulfide bridges in orange.. The intramembranous sequence is highlighted in grey. For the homodimerisation patch of β49-55 residues are marked with @ at the bottom of the sequence and highlighted in light green.

Fig S2. HLA-DR4 amino acid sequences.


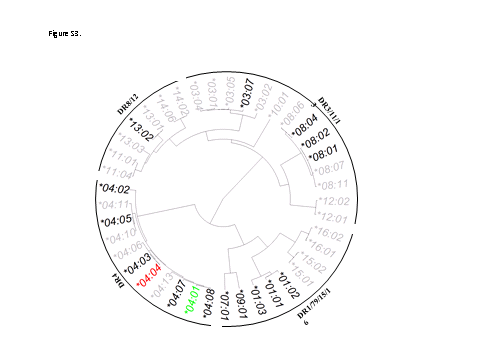


Figure S3. A fan-shaped hierarchical representation of all observed DRB1 alleles in TEDDY is obtained through phylogenic analysis of sequence similarities, and clusters of alleles are named by alleles in corresponding clusters.


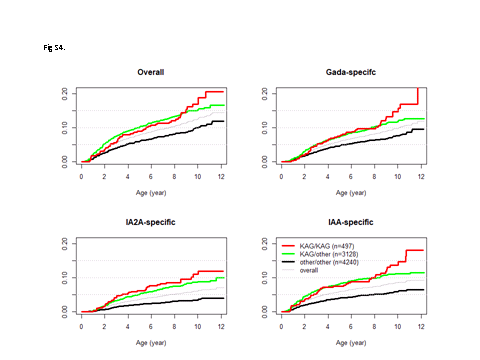


Fig S4. Incidence curves of overall, GADA-specific, IA2A-specific and IAA-specific seroconversion for carriers of homozygous motif “KAG/KAG” (red line), heterozygote “KAG/OTH” (green), and all others “OTH/OTH” (black; OTH represents all of other motifs combined than “KAG”). Both incidence curves among carriers of “KAG” are significantly greater than that of OTH/OTH (see Table 10). A thin gray line represents the average incidence curve.
