# Supplementary material for: The KAG motif of HLA-DRB1 (β71, β74, β86) predicts seroconversion and development of type 1 diabetes
Source: eBioMedicine. 2021 Jun 19;69:103431. doi: 10.1016/j.ebiom.2021.103431 (PMC8220560; doi:10.1016/j.ebiom.2021.103431)
Supplement: Supplementary file 2 [file mmc2.docx]

Table S1. Distribution of family history, gender, and study sites among 7865 subjects in TEDDY cohort

|  |  | **General Population** | **First Degree Relatives** |
| --- | --- | --- | --- |
|  | n= | 6982 (100) | 883 (100) |
| **Gender** | Girl | 3409 (49) | 452 (51) |
|  | Boy | 3573 (51) | 431 (49) |
|  |  |  |  |
| **Sites** | Finland | 1579 (23) | 151 (17) |
|  | Germany | 340 (5) | 202 (23) |
|  | Sweden | 2179 (31) | 161 (18) |
|  | US | 2884 (41) | 369 (42) |
|  |  |  |  |
| **Birth** | 2004 | 216 (3) | 25 (3) |
|  | 2005 | 943 (14) | 115 (13) |
|  | 2006 | 1258 (18) | 154 (17) |
|  | 2007 | 1454 (21) | 211 (24) |
|  | 2008 | 1427 (20) | 178 (20) |
|  | 2009 | 1468 (21) | 180 (20) |
|  | 2010 | 216 (3) | 20 (2) |
|  |  |  |  |
| **Season** | Winter | 1775 (25) | 233 (26) |
|  | Spring | 1663 (24) | 201 (23) |
|  | Summer | 1766 (25) | 225 (25) |
|  | Fall | 1778 (25) | 224 (25) |

Table S2A. Allelic/haplotypic frequencies of DRB1 across four countries. DR4 subtypes were highlighted.

|  | DRB1 | **US** | **Sweden** | **Finland** | **Germany** | **Total** |
| --- | --- | --- | --- | --- | --- | --- |
|  | n= | 1730 (100) | 542 (100) | 2340 (100) | 3253 (100) | 7865 |
| 1 | *01:01 | 38 (1) | 20 (0) | 42 (1) | 39 (4) | 139 |
| 2 | *01:02 | 9 (0) | 1 (0) |  | 2 (0) | 12 |
| 3 | *01:03 | 8 (0) |  |  | 5 (0) | 13 |
| 4 | *03:01 | 2811 (43) | 1973 (42) | 1085 (31) | 444 (41) | 6313 |
| 5 | *03:02 | 9 (0) |  |  |  | 9 |
| 6 | *04:01 | 1327 (20) | 1420 (30) | 1068 (31) | 306 (28) | 4121 |
| 7 | *04:02 | 249 (4) | 84 (2) | 13 (0) | 46 (4) | 392 |
| 8 | *04:03 | 10 (0) | 3 (0) | 14 (0) | 6 (1) | 33 |
| 9 | *04:04 | 1124 (17) | 763 (16) | 628 (18) | 111 (10) | 2626 |
| 10 | *04:05 | 214 (3) | 53 (1) | 14 (0) | 28 (3) | 309 |
| 11 | *04:07 | 194 (3) | 12 (0) | 6 (0) | 2 (0) | 214 |
| 12 | *04:08 | 14 (0) | 12 (0) | 2 (0) | 11 (1) | 39 |
| 13 | *04:10 | 3 (0) | 1 (0) | 1 (0) |  | 5 |
| 14 | *07:01 | 11 (0) | 1 (0) | 5 (0) | 2 (0) | 19 |
| 15 | *08:01 | 251 (4) | 284 (6) | 522 (15) | 41 (4) | 1098 |
| 16 | *08:02 | 156 (2) | 16 (0) | 26 (1) | 3 (0) | 201 |
| 17 | *08:04 | 21 (0) | 5 (0) | 1 (0) | 5 (0) | 32 |
| 18 | *09:01 | 10 (0) | 5 (0) | 18 (1) | 6 (1) | 39 |
| 19 | *13:01 | 2 (0) | 1 (0) | 3 (0) | 1 (0) | 7 |
| 20 | *13:02 | 22 (0) | 15 (0) | 7 (0) | 16 (1) | 60 |
| 21 | *15:01 | 2 (0) | 4 (0) |  |  | 6 |
| 22 | *15:02 |  | 2 (0) | 1 (0) | 4 (0) | 7 |
|  | rare(<5) | 21 (0) | 5 (0) | 4 (0) | 6 (1) | 36 |

Table S2B. Allelic/haplotypic frequencies of DQA1-DQB1 across four countries. DR4 subtypes were highlighted.

|  | DQA1-DQB1 | **US** | **Sweden** | **Finland** | **Germany** | **Total** |
| --- | --- | --- | --- | --- | --- | --- |
|  | n= | 1730 (100) | 542 (100) | 2340 (100) | 3253 (100) | 7865 |
| 1 | *01:01-*05:01 | 56 (1) | 21 (0) | 42 (1) | 47 (4) | 166 |
| 2 | *01:02-*06:02 | 2 (0) | 4 (0) |  |  | 6 |
| 3 | *01:02-*06:04 | 21 (0) | 14 (0) | 8 (0) | 16 (1) | 59 |
| 4 | *01:03-*06:01 |  | 2 (0) | 2 (0) | 4 (0) | 8 |
| 5 | *02:01-*02:01 | 8 (0) |  | 4 (0) |  | 12 |
| 6 | *03:01-*02:01 | 4 (0) | 1 (0) | 1 (0) | 1 (0) | 7 |
| 7 | *03:01-*03:01 | 2 (0) | 2 (0) | 1 (0) | 2 (0) | 7 |
| 8 | *03:01-*03:02 | 3122 (48) | 2338 (50) | 1735 (50) | 500 (46) | 7695 |
| 9 | *03:01-*03:03 | 8 (0) | 3 (0) | 20 (1) | 6 (1) | 37 |
| 10 | *03:01-*03:04 | 14 (0) | 11 (0) | 6 (0) | 10 (1) | 41 |
| 11 | *04:01-*04:02 | 438 (7) | 304 (6) | 550 (16) | 49 (5) | 1341 |
| 12 | *05:01-*02:01 | 2817 (43) | 1976 (42) | 1085 (31) | 444 (41) | 6322 |
| 13 | *05:01-*03:01 | 3 (0) |  | 2 (0) |  | 5 |
|  | rare(<5) | 11 (0) | 4 (0) | 4 (0) | 5 (0) | 24 |

Table S2C. Allelic/haplotypic frequencies of DRB1, DQA1 and DQB1 across four countries. DR4 subtypes were highlighted.

| ID | DRB1-DQA1-DQB1 | **US** | **Sweden** | **Finland** | **Germany** | **Total** |
| --- | --- | --- | --- | --- | --- | --- |
|  | n= | 1730 (100) | 542 (100) | 2340 (100) | 3253 (100) | 7865 |
| 1 | *01:01-*01:01-*05:01 | 38 (1) | 20 (0) | 42 (1) | 39 (4) | 139 |
| 2 | *01:02-*01:01-*05:01 | 9 (0) | 1 (0) |  | 2 (0) | 12 |
| 3 | *01:03-*01:01-*05:01 | 8 (0) |  |  | 5 (0) | 13 |
| 4 | *03:01-*05:01-*02:01 | 2811 (43) | 1973 (42) | 1085 (31) | 444 (41) | 6313 |
| 5 | *03:02-*04:01-*04:02 | 9 (0) |  |  |  | 9 |
| 6 | *04:01-*03:01-*03:01 | 2 (0) | 2 (0) | 1 (0) |  | 5 |
| 7 | *04:01-*03:01-*03:02 | 1322 (20) | 1418 (30) | 1066 (31) | 306 (28) | 4112 |
| 8 | *04:02-*03:01-*03:02 | 249 (4) | 84 (2) | 13 (0) | 46 (4) | 392 |
| 9 | *04:03-*03:01-*03:02 | 10 (0) | 3 (0) | 13 (0) | 5 (0) | 31 |
| 10 | *04:04-*03:01-*03:02 | 1123 (17) | 763 (16) | 623 (18) | 110 (10) | 2619 |
| 11 | *04:04-*03:01-*03:04 |  |  | 5 (0) | 1 (0) | 6 |
| 12 | *04:05-*03:01-*03:02 | 210 (3) | 52 (1) | 13 (0) | 27 (2) | 302 |
| 13 | *04:07-*03:01-*03:02 | 192 (3) | 12 (0) | 6 (0) | 2 (0) | 212 |
| 14 | *04:08-*03:01-*03:02 | 5 (0) | 1 (0) | 1 (0) | 2 (0) | 9 |
| 15 | *04:08-*03:01-*03:04 | 9 (0) | 11 (0) | 1 (0) | 7 (1) | 28 |
| 16 | *07:01-*02:01-*02:01 | 8 (0) |  | 4 (0) |  | 12 |
| 17 | *08:01-*04:01-*04:02 | 251 (4) | 282 (6) | 522 (15) | 41 (4) | 1096 |
| 18 | *08:02-*04:01-*04:02 | 155 (2) | 16 (0) | 26 (1) | 3 (0) | 200 |
| 19 | *08:04-*04:01-*04:02 | 21 (0) | 5 (0) | 1 (0) | 5 (0) | 32 |
| 20 | *09:01-*03:01-*03:03 | 8 (0) | 3 (0) | 18 (1) | 6 (1) | 35 |
| 21 | *13:02-*01:02-*06:04 | 20 (0) | 14 (0) | 7 (0) | 15 (1) | 56 |
| 22 | *15:01-*01:02-*06:02 | 2 (0) | 4 (0) |  |  | 6 |
| 23 | *15:02-*01:03-*06:01 |  | 2 (0) | 1 (0) | 4 (0) | 7 |
|  | rare(<5) | 44 (1) | 14 (0) | 12 (0) | 14 (1) | 84 |

Table S3. Seroconversion associations with study sites (US vs Europe), family history (First Degree Relative vs General Population), and gender (boy vs girl)

|  | **Coef** | **HR** | **SE** | **Z.score** | **Pvalue** |
| --- | --- | --- | --- | --- | --- |
|  | Univariate Association | | | | |
| US vs Europe | -0.27 | 0.77 | 0.08 | -3.35 | 8.08E-04 |
| First Degree Relative vs General Population | 0.63 | 1.88 | 0.09 | 6.73 | 1.72E-11 |
| Boy vs Girl | 0.15 | 1.17 | 0.07 | 2.06 | 3.99E-02 |
|  | Multivariate Association | | | | |
| US vs Europe | -0.27 | 0.76 | 0.08 | -3.45 | 5.53E-04 |
| First Degree Relative vs General Population | 0.64 | 1.90 | 0.09 | 6.82 | 9.15E-12 |
| Boy vs Girl | 0.16 | 1.18 | 0.07 | 2.16 | 3.11E-02 |
