# Supplementary material for: The KAG motif of HLA-DRB1 (β71, β74, β86) predicts seroconversion and development of type 1 diabetes
Source: eBioMedicine. 2021 Jun 19;69:103431. doi: 10.1016/j.ebiom.2021.103431 (PMC8220560; doi:10.1016/j.ebiom.2021.103431)
Supplement: Supplementary file 3 [file mmc3.docx]

**BDD Study Group Supplemental file**

A

nnelie Carlsson, Department of Clinical Sciences, Lund University, Lund, Sweden

Helena Elding Larsson, Department of Clinical Sciences, Lund University, Malmö, Sweden

Gun Forsander, Institute of Clinical Sciences, University of Gothenburg, Gothenburg, Sweden

Åke Lernmark, Department of Clinical Sciences, Lund University, Malmö, Sweden

Johnny Ludvigsson, Department of Clinical and Experimental Sciences, Linköping University, Linköping, Sweden

Claude Marcus, Department of Clinical Science, Intervention and Technology, Karolinska Institutet, Stockholm, Sweden

Martina Persson, Department of Medicine, Clinical Epidemiology, Karolinska University Hospital, Stockholm, Sweden

Auste Pundziute-Lyckå, Department of Pediatrics, Queen Silvi Children’s Hoaspital, Gotheburg, Sweden

Ulf Samuelsson, Department of Clinical and Experimental Medicine, Linköping University, Linköping, Sweden

Karin Åkesson, Department of Pediatrics, Ryhov County Hospital, Jönköping, Sweden
