# Supplementary material for: The KAG motif of HLA-DRB1 (β71, β74, β86) predicts seroconversion and development of type 1 diabetes
Source: eBioMedicine. 2021 Jun 19;69:103431. doi: 10.1016/j.ebiom.2021.103431 (PMC8220560; doi:10.1016/j.ebiom.2021.103431)
Supplement: Supplementary file 4 [file mmc4.docx]

| **First name** | **Surename** | **Department** | **Institution** | **Address** | **City** |
| --- | --- | --- | --- | --- | --- |
| Annelie | Carlsson | Department of Clinical Sciences | Lund University | SUS | Malmö |
| Åke | Lernmark | Department of Clinical Sciences | Lund University | SUS | Malmö |
| Claude | Marcus | Dep.of Clinical Science Intervention and Technology | Karolinska Institute |  | Stockholm |
| Johnny | Ludvigsson | Dep. of Clinical and Experimental Medicine | Linköping University |  | Linköping |
| Gun | Forsander | Institute of Clinical Sciences | University of Gothenburg |  | Gothenburg |
| Helena | Elding-Larsson | Department of Clinical Sciences | Lund University | SUS | Malmö |
| Ulf | Samuelsson | Dep. of Clinical and Experimental Medicine | Linköping University |  | Linköping |
| Martina | Persson | Department of Medicine, Clinical Epidemiology | Karolinska University Hospital |  | Stockholm |
| Karin | Åkesson | Department of Pediatrics | Ryhov County Hospital |  | Jönköping |
| Auste | Pundziute-Lycka | Department of Pediatrics | Queen Silvia Children's Hospital |  | Gothenburg |

**BDD steering committee**
